# The effects of manipulating choice on children’s enjoyment and performance in a reading task

**DOI:** 10.1007/s12144-025-07685-3

**Published:** 2025-03-17

**Authors:** Lisa Fridkin, Jane Hurry

**Affiliations:** 1 Social Research Institute, UCL, Institute of Education, London, UK; 2 Department for Psychology and Human Development, UCL, Institute of Education, London, UK

**Keywords:** Situational interest, Intrinsic motivation, Reading performance, Enjoyment, Choice

## Abstract

This study investigates effects of choice in a reading comprehension task. It hypothesises that choice will act as a trigger for situational interest, impacting engagement with the reading text, and will therefore improve children’s performance in a reading comprehension task, and promote higher levels of enjoyment for that task. Participants were 110 Grade 3 pupils (61 boys, 49 girls). Reading comprehension performance and task enjoyment were measured in a cross-over, repeated measures design where children were either allocated a short story or offered a perceived choice of story to read. In fact, all children read the same story in each condition. Reading comprehension scores and post-test reported enjoyment scores were gathered and analysed. Choice was found to significantly affect comprehension scores (Cohen’s *d* = 0.52) and reported task enjoyment (Cohen’s *d* = 0.23), indicating that choice impacts engagement with a reading text. Effects did not vary by gender or ability. Reading motivation promoted by situational interest may play an important role in reading comprehension and choice may be an effective trigger for situational interest in a reading task and a powerful intrinsic motivator. Situational interest, triggered by choice, may be effective in raising enjoyment levels for a reading task.

## Introduction

There are strong established links between academic achievement and motivation which are also recognised in reading development (Deci & Ryan, 1985; Guthrie et al., 2012; Schiefele et al. 2012). However, the association between reading motivation and reading development receives less attention (Cambria & Guthrie, 2010) than the cognitive skills that contribute to reading comprehension skill (Cremin & Scholes, 2024; Wigfield et al., 2014). Longitudinal evidence suggests a positive, reciprocal relationship between intrinsic reading motivation and reading comprehension, most securely from the direction of comprehension to motivation, whilst extrinsic motivation has been found to have a negative association with subsequent reading (Becker et al. 2010; Hebbecker et al., 2019; Schiefele et al., 2016). Despite some recognition that reading comprehension skills cannot be fully understood without considering the role played by motivation (Sullivan & Brown, 2015; Wigfield et al., 2004; Wilkinson et al., 2020), there is a lack of research investigating the processes mediating links between intrinsic motivation and task-related reading comprehension. Situational interest is understood to impact intrinsic motivation in-the-moment and effect both affective and cognitive responses to a stimulus. In this way, effective manipulation of situational interest in a reading task might be expected to elicit an improvement in both task enjoyment and performance. The current study set out to investigate whether choice, a recognised trigger for situational interest, can stimulate children’s immediate affective and cognitive responses in a reading task.

This research experiments with the effects of choice, a central, well-recognised motivational variable, in a reading comprehension task. Choice is recognised as key for achieving autonomy in the primary theory of intrinsic motivation, self-determination theory (Ryan & Deci, 2000), has been identified as one of the five dimensions of reading motivation (Taboada et al., 2009) and has been described as an important potential motivational trigger within the construct of interest (e.g., Cordova & Lepper, 1996; Guo & Fryer, 2024; Schraw et al., 1998; Wigfield et al., 2014), and interest theory (Hidi & Renninger, 2006; Renninger & Hidi, 2022). This study uses an experimental design which allows for focus on specific areas of motivation and to address issues of causality, therefore overcoming prior criticisms of research evidence as difficult to interpret because of its correlational nature or because engaging practices were tested in combination with other elements (Shanahan et al., 2010). Furthermore, manipulating choice in an experimental design provides the advantage of avoiding reliance on problematic subjective accounts of participants’ motivation, which may be particularly relevant with young children.

### Motivation and situational interest

Theories concerning intrinsic and extrinsic motivation examine why we would want to focus attention and expend effort on a particular activity. This study focuses on intrinsic motivation and more specifically on interest. Whilst extrinsic rewards do increase desired behaviours, it is argued that they undermine intrinsic motivation (see Deci et al., 1999 for a meta-analysis of studies), although evidence now suggests a more complex picture, acknowledging the value of extrinsic motivators in low interest tasks and in the development of intrinsic interest (Hidi & Harackiewicz, 2000; Morris et al., 2022). Nonetheless, there is a consensus that intrinsic motivation is important for academic achievement, not least because it is self-generating, independent of external actions.

Intrinsic motivation is intimately linked with interest where changes in levels of intrinsically motivated effort may be facilitated by either pre-existing topic interest (also referred to as personal interest) or situational interest (Hidi, 1990; Hidi & Renninger, 2006; Renninger & Hidi, 2022). Situational interest is described as a passing reaction to a stimulus in the environment which elicits an unconscious, heightened engagement in that specific context (Hidi & Renninger, 2006; Krapp, 2002). This is of practical significance in reading development because it proposes that environmental features, which educators may control, can alter how we engage with a stimulus: where it is managed successfully, it can increase levels of attention, effort and enjoyment (Hidi & Renninger, 2006; Renninger & Hidi, 2022; Renninger et al., 2024) Situational interest is well-suited to the investigation of the effects of interest on reading as, unlike topic interest, it is not associated with background topic knowledge and vocabulary, both of which support reading comprehension and therefore complicate the interpretation of the relationship between topic interest and reading performance. It is also of theoretical importance, as although it cannot account for immediate increases in long-term motivation and achievement, it is proposed as the first stage in the development of interest (Hidi & Harackiewicz, 2000), providing a springboard for positive and enjoyable reading experiences (Cambria & Guthrie, 2010; Wigfield et al., 2014): it is well documented that reading for pleasure positively impacts reading achievement (e.g., Clark & Teravainen-Goff, 2020; Cremin & Scholes, 2024; Heyne et al., 2023; Sullivan & Brown, 2015). Indeed, Hidi and Renninger (2006), propose that situational interest brings about a unique interplay between affective and cognitive elements resulting in effortless yet enhanced engagement. In this way, triggers for situational interest may promote both reading success and reading enjoyment, and may therefore be a useful factor in reading development. Establishing which variables trigger such a response, and how effective they might be in improving reading, is of considerable practical and theoretical significance, yet currently under-researched in the domain of reading development.

### Choice

Choice has long been recognised as an important and powerful motivational variable (e.g., deCharms, 1968; Lewin, 1952; Patall, 2012) and may be highly effective in educational settings, successfully impacting intrinsic motivation and interest levels (e.g. Asher & Harackiewicz, 2024; Tegmark et al., 2022; Wang & Eccles, 2013). Despite differing theoretical perspectives (e.g., Guthrie & Wigfield, 2000; Hidi & Renninger, 2006; Ryan & Deci, 2000) on the underpinnings of choice, its role as a motivational factor is consistently understood in broadly similar terms: the effects of choice can stimulate an increase in intrinsic motivation. It has also been shown to lead to improved performance in secondary-school age children in a learning task (Schneider et al., 2018). In the domain of reading, Concept Oriented Reading Instruction (CORI), found to enhance reading motivation and performance (Guthrie et al., 2007), advocates meaningful choices to stimulate situational interest (Wigfield et al., 2004), although the inclusion of a range of strategies and motivational practices prevents clear empirical evidence for the role of choice alone.

Empirical studies reflect the tenacity of choice as a motivational tool but also its fragility, insofar as the evidence produced is thinly stretched over domains, activity, age groups and can be inconsistent (e.g., Guo & Fryer, 2024; MacNaul et al., 2021; Schraw et al., 1998). Whilst there is agreement from many investigations that choice positively affects motivation and consequently positively impacts performance in the short term (e.g., average Cohen’s *d* = 0.36 in meta-analysis of 41 studies, Patall et al., 2008), they also inform us that the mechanisms surrounding choice are sensitive (e.g., Cordova & Lepper, 1996; Flowerday & Shell, 2015; Guthrie, Wigfield & colleagues; Patall, 2012, 2013; Patall et al., 2010; Zuckerman et al. 1978). Whether choice will have a positive, neutral or negative effect on motivation has been shown to be dependent on a variety of factors: number of choices offered (too many can be overwhelming); the way in which choice is presented; the characteristics of the individual being offered the choice (prior knowledge, culture and personal interests); the characteristics of the choice (if it is meaningful) (Patall, 2012). The current study addresses these issues through a controlled design that specifically takes account of these factors and furthers our understanding of their effects on choice.

#### Meaningful choice

Understanding meaningful choice is illustrated in evidence provided by Iyengar and Lepper (1999) who found that elementary school children performed better in an anagram puzzle task that they chose compared to a task chosen for them. However, the impact of choice was moderated by cultural difference. Asian American school children’s performance was stronger when choices were made for them by a significant figure (their mother) whereas personal choice was more effective for Anglo American children. Similarly, Rosenzweig and colleagues (2019), demonstrated that the effects of choice were mediated by the utility value (personal meaningfulness) of course materials. Thus, college students reported significantly higher interest for course content in a high-choice condition compared to a low-choice condition and control condition. Similarly, Asher and Harackiewicz (2024), with a study manipulating meaningful choice and utility value together, found that both contributed to promoting interest, and meaningful choice elicited higher reported engagement and attention in undergraduate students on a statistics course. McClung et al. (2019) also found effects in favour of meaningful choice, where children showed greater literacy achievement when they were given scope to select their reading materials compared to when the materials were directed by their teacher. This evidence compares to an experiment by Flowerday et al. (2004), intended to disentangle effects of choice from interest in what is chosen, where they found that choice had no effect on engagement or performance. Their study offered college students a blind choice of one task over another (completing an essay or crossword) in the form of selecting from two sealed envelopes containing the task. These findings may reflect the need for choice to be meaningful by offering personal connection, not just be an invitation to pick between two options (Katz & Assor, 2007). The current study therefore provides participants with an opportunity to make an informed and therefore meaningful choice– through reviews from children of a similar age– about which story they would like to read.

#### Characteristics of choice

Regarding the characteristics of the choice, choices may make a difference even if relatively trivial, if experienced by participants as meaningful (Patall, 2012). For example, Cordova and Lepper (1996) examined the effects of choice of what they define as “instructionally irrelevant aspects of the task” among elementary school children involved in an educational computer activity. In their choice condition, participants could choose features such as the icon representing them on the game board, the name of their spaceship, the name of their opponents, and their starting point of two shortcuts. Choice significantly enhanced learning and motivation.

Renninger and Hidi (2011, 2022) propose that interest promotes an affective yet unconscious response and they therefore caution against the validity of self-report measures of interest. This is likely to be particularly true for children, who are typically less aware of internal processes. Experimental manipulation of situational interest using variables such as choice, which have a good track record of enhancing motivation, avoids reliance on self-report to investigate the processes that link motivation to reading comprehension.

#### Age, gender and ability

Further variables such as age, gender and ability may also interact with these different factors and mediate any potential effects. For example, although it has been suggested that choice is more effective as a motivational variable amongst children rather than older students (Flowerday & Schraw, 2000; Hidi & Anderson, 1992; Patall et al., 2008) the majority of studies have been conducted with middle school and college students or adults, and therefore this claim lacks substantiation. Indeed, it has also been acknowledged that there is a need to investigate the role of choice across age groups (Flowerday & Schraw, 2003). Although the influential work through CORI of Guthrie, Wigfield and colleagues (Guthrie & Wigfield, 2000) proposes choice as an element of a motivational programme with both elementary and middle school learners, their work does not isolate the effectiveness of choice independently.

Reading development and reading ability research commonly highlight gender differences, and this has also emerged in studies investigating the influence of interest and motivation (e.g., Bernstein, 1955; Oakhill & Petrides, 2007), where boys were more susceptible to the effects of motivational triggers than girls, and boys more likely to perform better where there is text-based interest (Lepper et al., 2022). A recent meta-analysis of 39 studies of motivation intervention and reading (van der Sande et al., 2023) found motivation intervention had a greater effect on boys’ reading motivation compared to girls, but not on their reading comprehension. There is little evidence reporting whether such differential effect occurs in experiments investigating choice. There are similar issues around the effects of interest and choice on different ability levels: teachers commonly report that choice is believed to have a greater motivational impact on low ability and low interest students (Schraw et al., 2001); and the interestingness of a reading comprehension task (editing versus a more interesting detective task) had greater impact on the performance of poor compared to good comprehenders aged 8 to 9 years (deSousa & Oakhill, 1996). In contrast, Guthrie and colleagues found that all ability groups performed better on post-test measures, including reading comprehension, compared to the control group following a 12-week CORI intervention (Guthrie et al., 2009). Adding to this inconsistency, van der Sande and colleagues (van der Sande et al., 2023) found motivation intervention had a greater effect on typical readers than those below average.

### Study aims

It has been proposed that situational interest is triggered and sustained by an environmental factor outside of the individual (Hidi & Renninger, 2006; Krapp, 2002) yet few studies have expressly investigated the direct effects of situational interest arising from choice on academic performance or on reading comprehension in children. Understanding the diverse nature of how choice operates as a motivational tool is challenging both theoretically and experimentally.

To overcome the criticism of over-use of self-report in measuring interest (Renninger & Bachrach, 2015), motivation in the form of choice is experimentally manipulated rather than measured, and its effect on reading comprehension and task enjoyment measured as outcomes of this manipulation.

In this study a perceived yet meaningful choice is presented to participants using a within subjects experimental design and measuring the impact of choice versus no choice in a reading comprehension task. Choice is anticipated to behave as a motivational variable, as described by theories of situational interest, where successfully triggered situational interest elicits increased effort, attention and enjoyment in an activity. It is therefore hypothesised that this manipulation will be evident in: (1) improved reading comprehension scores in the experimental (choice) compared to the control (no choice) condition and; (2) in higher reported enjoyment for the text read in the experimental condition compared to the control.

Prior research and theory indicate gender and ability differences in reading comprehension performance, motivation and interest in reading texts, with girls typically outperforming boys, boys more likely to be influenced by interest (Bernstein, 1955; Oakhill & Petrides, 2007), and a mixed-picture on pupils’ reading ability (deSousa & Oakhill, 1996; Guthrie et al., 2009; Logan et al., 2011; van der Sande et al., 2023). The following secondary hypothesis is therefore also investigated: that there will be a difference in reading comprehension scores and enjoyment across the two conditions which will be moderated by gender, where choice will have a greater effect on boys. Further, the role of reading ability as a moderator will also be examined.

## Method

### Participants

Participants were drawn from Grade 3 (age 8–9) of two two-form entry mainstream schools, in south-east England. Further information about school characteristics is provided in Appendix A.

A total of 110 participants (61 boys, 49 girls) were included in the final sample, 54 from School 1 and 56 from School 2. There was an above average percentage of 46% of children for whom English was a second language (ESL), although only 1 of these children was in the early stages of learning English; 13% of the children, compared with 15% nationally, were eligible for Pupil Premium (a measure of social disadvantage applied to all English state schools); 13% of children had a special educational need, compared with 15% nationally. The only exclusions from the sample were 10 pupils who had not completed both comprehension exercises due to school absence. These 10 pupils did not significantly differ from the final sample of 110 on either gender or reading ability.

Parental consent for children’s participation in the study was given for all children, and children also assented to taking part. Children were told that their participation was voluntary and that they could stop their participation at any point. The project received ethical approval through the university ethics committee.

### Design

All children completed a pre-test to assess reading ability. Reading test scores were compared with teacher reading assessment scores in order to identify potential anomalies and then used to rank the children within their class group. Children were matched by ability and gender within each class in each school and randomly assigned to one of two groups for text order. A repeated measures design was used where Group 1 completed the experimental condition (perceived choice of story to read and reading comprehension questions) followed by the control condition (allocated story to read and reading comprehension questions) and Group 2 completed the control followed by experimental condition. In order to control for order and story effects, a cross-over design was employed so that both texts were used in both conditions as set out in Table 1 below. Enjoyment Questionnaires (see Appendix B for a sample of questions) were completed by all children immediately following each reading comprehension task to measure enjoyment of each activity.

**Table 1:** Illustration of condition and story by group and school

School I		School II	
Group 1		Group 1	
Experimental Condition	Choice: Story 1	Experimental Condition	Choice: Story 2
Control Condition	No Choice: Story 2	Control Condition	No Choice: Story 1
Group 2		Group 2	
Control Condition	No Choice: Story 1	Control Condition	No Choice: Story 2
Experimental Condition	Choice: Story 2	Experimental Condition	Choice: Story 1

### Materials

#### Pre-test for reading ability

Reading comprehension ability was assessed using a standardised test, New Group Reading Test (Burge et al., 2010). Tests 2 A and 2B, designed for children aged 6:00 to 10:05 were used. The tests comprise two sections. The first section comprises 20 items with a multiple-choice format where it is necessary to select the word that is the best fit to complete the sentence. The second section comprises three different passages with accompanying multiple-choice questions which are a mixture of context comprehension questions and reading comprehension questions. Raw scores range from 0 to 48, standard scores have a mean of 100 and a standard deviation of 15. The tests evaluate vocabulary, grammatical knowledge, inference skills and deduction skills.

The test provides high levels of reliability with Cronbach’s alpha reported at above 0.9 for both tests. Validity is based on high levels of reliability, adequate representation of the construct of reading and elimination of irrelevant factors in accordance with the arguments of William (2008). These are demonstrated through the high levels of test reliability, supporting evidence that the test effectively assesses the construct of reading, and a format where writing is eliminated through the sole use of multiple-choice questions.

#### Reading comprehension texts

In order to assess reading comprehension performance, two short stories were written and matched for word length and difficulty using a readability formula tool (Spache, 1953). The main text section for Story 1 about a child’s birthday, comprised 627 words and 7 pictures and for Story 2, about a surprise holiday, 682 words and 7 pictures. To allow pupils a perceived choice, in line with the experimental design requirements, two alternative cover pages, each with an individual design and title together with two different first pages were created for each story (‘Something’s going on’ or ‘Just another Ordinary Day’ for Story 1 and ‘Wishing on a Star’ or ‘A Snowy Adventure’ for Story 2). The four first pages all comprised 57 to 83 words and 1 picture and were all assigned a Grade Level 4, reading age 8–9 years and a ‘very easy to read’ text difficulty using the same readability assessment procedure outlined for the storybooks. The content from these pages did not relate to the comprehension questions. Thus a total of four cover pages, four first pages and two stories were written. This design enabled a perceived choice to be offered to participants where the main text that related to the comprehension questions for each of the two stories was identical.

Story Reviews. A total of four short story reviews were written (two per story) that gave generic and favourable opinions of each story with no reference made to any story content. They were comparable in length and style as evaluated through the pilot study and by an experienced primary school teacher. Two of the reviews were attributed to girls and two to boys, described by a first name and age. The reviews were based on comments made by the children who took part in the Pilot Study. The reviews were paired so that one from each sex was assigned to each text of the choice condition storybook. The same reviews were used for both stories.

Comprehension Questions. Reading comprehension questions were developed for each story using Key Stage 2 English Statutory Assessment Tests (SATs) questions as a model and included a well-balanced combination of both literal and inferential questions and response type (multiple choice or written response). Eighteen questions were reviewed by an experienced primary school teacher and trialled in a pilot study with one Grade 3 class to ensure they were fit for purpose. Taking into account teacher and child comments and the results of a Cronbach’s alpha test by item for each story, the final 12-item set of questions achieved Cronbach’s alpha of 0.84 (Story 1) and 0.75 (Story 2). The questions selected for each story reflected the same format and question type and made no reference to the content of the different first pages. The 12 items for each set of questions were scored either 0 or 1, and the maximum score was 14. The comprehension scale had a Cronbach’s alpha value of 0.73 (Story 1 1) and 0.55 (Story 2), indicating an acceptable level of reliability for Story 1 but a somewhat weak level for Story 2. Stories 1 and 2 were of comparable level of difficulty (Story 1, *M* = 6.71, *SD* = 2.81; Story 2, *M* = 6.67, *SD* = 2.69). Concurrent validity was measured by calculating Pearson’s r correlation coefficient for the three measures of reading (comprehension scores in the control condition for each story and NGRT raw scores). The correlations were: *r* = .498, between the two sets of comprehension scores; *r* = .603 between Story 1 comprehension scores and NGRT raw scores and *r* = .574 between Story 2 comprehension scores and NGRT raw scores indicating satisfactory though moderate correlation strength according to Cohen, 1988.

Enjoyment Questionnaire. Enjoyment for each story after reading was assessed using a 14-item questionnaire, answered using a 4-point Likert scale ranging from 1 (very different from you) to 4 (a lot like you). This questionnaire was designed in order to assess the extent to which participants enjoyed each story immediately after reading. Questions asked about enjoyment explicitly (e.g. I really enjoyed this story), about a feeling of competence (e.g. The questions were easy), where competence is considered an important element of enjoyment in the context of learning (Ainley & Hidi, 2014), and about a willingness to continue with the activity (e.g. I found it difficult to keep going), which presents a further recognised dimension of enjoyment (Reeve, 1989). The questions were reviewed by a primary school teacher before being trialled in a pilot study to ensure they were fit for purpose. Internal consistency for the questionnaire was analysed by calculating Cronbach’s alpha, giving an overall value of 0.718, indicating an acceptable level of reliability.

### Procedure

This study was granted ethical approval by the university ethics committee. All testing took place in the participants’ classrooms during a morning session at school. Prior to the testing phase, all participants completed the New Group Reading Test (NGRT, Burge et al., 2010). For the main testing, in each school participants were ranked by gender and ability (as assessed by the NGRT raw score) and then randomly assigned to one of two groups where Group 1 carried out the experimental condition in session 1 and the control in session 2 and Group 2 the control condition in session 1 and the experimental condition in session 2. In School 1 children in both groups received Story 1 in session 1 and Story 2 in session 2, in School 2 story order was reversed, with Story 2 in session 1 and Story 1 in session 2. All sessions were between 24 and 72 h apart.

For the experimental condition, the participants were handed two C4 envelopes with the illustrated cover and first page of a story, and two story reviews stapled to the front. Participants were asked to read through this material and use it to help them decide which story they would then most like to read. Participants were then asked to read their chosen story and answer the accompanying question sheet, both found in the selected envelope. In fact the story in each envelope was the same story with a different front cover and first page. No support was given to participants during the exercise but they were allowed to refer to the story throughout the comprehension task. There was no time limit imposed: participants were asked to read their current reading book to allow all classmates to finish. On completion, all participants completed the Enjoyment Questionnaire, where the statements were read aloud by the researcher and participants were given an opportunity to select their response for each item. For the control condition, participants were allocated a story to read with a set of comprehension questions. All other procedures were identical to those in the experimental condition.

A full debrief was given after both sessions were completed that made participants aware that there had only been a perceived choice in the experimental condition. The reasons for this were explained and participants were encouraged to ask questions.

## Results

Mean pre-test standard scores for NGRT for all participants are set out by class and gender in Table 2 below. There was no significant difference between mean pre-test reading standard scores by gender (boys *M* = 99.00, *SD* = 12.51; girls *M* = 103.23, *SD* = 11,50; *t* (105) = −1.81, *p* = .073; Cohen’s *d* = 0.35). There was no significant difference in pre-test reading score between children who received the no-choice condition first and those who received the choice condition first (no-choice first *M* = 99.20, *SD* = 12.75; choice first *M* = 102.58, *SD* = 11.47; *t* (105) = −1.44, *p* = .154; Cohen’s *d* = 0.28).

**Table 2:** Mean score for NGRT pre-tests by class group and gender

		NGRT Standard Score		
	Class	*N*	Mean	Standard Deviation
School I	1	26	104.46	11.92
	2	28	102.46	12.47
School II	3	28	100.96	11.04
	4	28	96.43	12.62
	Total	110	100.84	12.21
	Gender			
	Boy	61	99.00	12.51
	Girl	49	103.23	11.50
	Total	110	100.84	12.21

*NGRT* New Group Reading Test

Measures of reading comprehension and enjoyment were not correlated, either in the no-choice *(r* = .097*)* or choice conditions (*r* = .001*)*.

### Effects of choice on reading comprehension task performance

Normality tests showed the reading comprehension scores followed a normal distribution (no-choice comprehension skewness = 0.188, kurtosis = − 0.140; choice comprehension skewness = − 0.222, kurtosis = 0.541). Therefore, to examine the extent to which having a choice might impact reading comprehension task performance, a paired t-test was conducted. Observation of means by condition indicated that reading comprehension raw scores were significantly higher for participants in the experimental condition (choice) *M* = 7.31, *SD* = 2.77, than in the control condition (no choice) *M* = 6.07, *SD* = 2.58, (*t*(109) = 5.41, *p* < .001, Cohen’s *d* = 0.52). Supporting the hypothesis in the expected direction, these results indicate that reading comprehension scores were significantly higher when having a perceived choice of story to read compared to being given a story to read.

### Differences by ability and gender on effects of choice on reading comprehension task performance

To test whether any effect of choice on reading comprehension was moderated by reading ability or gender, a reading comprehension difference score was calculated for each child by subtracting their score in the no-choice condition from their score in the choice condition. This variable captured the magnitude of the effect of choice on a child’s reading comprehension. Two linear regressions were then conducted with the reading comprehension difference score as the dependent variable and NGRT score and gender respectively as independent variables. Neither NGRT raw score or gender statistically significantly predicted reading comprehension difference score at the one-tailed level (*α* < 0.100) (NGRT, *B* = 0.045, *SE* = 0.027, *p* = .103; Gender, *B* = −0.095, *SE* = 0.462, *p* = .837. It is perhaps worth noting that the direction of relationship between reading ability (NGRT) and the effect of choice (comprehension difference score) was in the opposite direction to that hypothesized, that is, children with higher levels of pre-test reading showed a greater, though non-significant, benefit from choice than less strong readers. These results indicate that the effect of choice on comprehension scores was not moderated by ability level, as recorded by scores on NGRT, or by gender.

### Effects of choice on reading comprehension task enjoyment

Normality tests showed that the data for enjoyment scores followed a normal distribution (no-choice enjoyment skewness = − 0.332, kurtosis = − 0.363; choice enjoyment skewness = 0.108, kurtosis = − 0.650). Therefore to examine the extent to which having a choice might impact reading comprehension task enjoyment, a paired t-test was conducted. Observation of means by condition indicated that enjoyment scores were higher for participants in the experimental condition (choice) *M* = 40.25, *SD* = 7.59, than in the control condition (no choice) *M* = 38.47, *SD* = 7.88, (t(109) = 2.37, *p* < .020, Cohen’s *d* = 0.23). Supporting the hypothesis in the expected direction, these results indicate that reading enjoyment scores were significantly higher when having a perceived choice of story to read compared to being given a story to read.

### Differences by ability and gender on effects of choice on reading comprehension task enjoyment

To test whether the effect of choice on reading enjoyment was moderated by reading ability or gender, a reading enjoyment difference score was calculated for each child by subtracting their score in the no-choice condition from their score in the choice condition. This variable captured the magnitude of the effect of choice on a child’s reading enjoyment. Two linear regressions were then conducted with the reading enjoyment difference score as the dependent variable and NGRT raw score and gender as independent variables. Neither NGRT score nor gender predicted reading enjoyment difference score (NGRT, *B* = −0.069, *SE* = 0.091, *p* = .451; Gender, *B* = 0.738, *SE* = 1.575, *p* = .640). These results indicate that the effect of choice on enjoyment scores was not moderated by ability level, as recorded by scores on NGRT, or by gender.

The impact of choice on reading comprehension task performance was not mediated by enjoyment. There was no significant correlation between enjoyment difference score and reading comprehension difference score (*r* = .082), indicating that choice operated independently on task enjoyment and task performance.

## Discussion

The main purpose of this study was to investigate whether providing meaningful choice acts a trigger for situational interest and enhances children’s reading comprehension and enjoyment in a reading task. A secondary aim was to examine differential effects by ability and gender. The analyses demonstrated that choice of reading material does positively impact both reading comprehension task performance and enjoyment independently and that these results are not moderated by either ability or gender.

Choice had a statistically significant effect of medium magnitude (Cohen’s *d* = 0.52) on performance in a reading task, considerably above the hinge point of *d* = 0.40 proposed by Hattie (2008) as the mark of a truly effective intervention. Whilst effects in this study are linked to a single task, we posit that if such effects were repeated on multiple tasks, they would make a worthwhile contribution to the reading curriculum, comparable to skills instruction interventions. The perceived choice offered in this study is not proposed as a technique that should be repeatedly used in the classroom. However, it demonstrates both the potential of choice and the impact of operationalising situational interest. Further, by manipulating a perceived choice, the present study eliminates effects of confounding variables such as prior interest or knowledge, an identified problem with previous research in this area (Guo & Fryer, 2024). Where prior research has struggled to isolate the effects of choice, for example by including skills instruction alongside motivational support (McBreen & Savage, 2020; van der Sande et al., 2023), in this study changes in reading performance are solely the result of choice.

We conceptualise choice within the theoretical framework of situational interest, and as a trigger for situational interest, Phase 1 of Hidi and Renninger’s four phase model, eliciting short-term changes with the potential to lead to Phase 4, well-developed, individual interest (Hidi & Renninger, 2006). Developed personal interest is highly desirable for academic achievement (e.g., Hidi & Harackiewicz, 2000), encouraging children to read more and with greater engagement (Cremin & Scholes, 2024). This study did not explicitly measure interest but successfully manipulated choice to trigger situational interest. The findings therefore direct us to a potentially rich avenue to drive the development of interest.

Motivation research is criticised for relying heavily on the use of self-report as a measure (Krapp & Prenzel, 2011; Renninger & Bachrach, 2015). The limitations associated with this method are particularly apparent for situational interest which is described as an unconscious and affective response to a stimulus (Renninger & Hidi, 2016). Additionally, it carries particular challenges for young children who may lack the necessary level of self-awareness to evaluate their own sense of motivation. This study overcomes some of these challenges by measuring changes in reading comprehension performance as an outcome of the manipulation of choice.

Self-report was employed to measure enjoyment of the reading task, an element of the construct of interest. Participants reported higher levels of enjoyment when they chose the text, statistically significant but of small magnitude (Cohen’s *d* = 0.23). Reading enjoyment has a significant relationship with several positive reading behaviours, such as increases in reading frequency and reading amount, which are associated with increases in reading achievement (Baker & Wigfield, 1999; Torppa et al., 2020). However, in our study, choice acted independently on children’s enjoyment and their reading comprehension, there was no evidence that its effect on reading comprehension was mediated by enjoyment as hypothesised. Whilst choice promoted higher levels of reading enjoyment for participants, they may not always be aware of this affective response, consistent with the theoretical link to situational interest (Hidi & Renninger, 2006). In a subsequent study applying similar methods to those used in the current study, the effect of choice on young children’s reading comprehension was replicated, both for neurotypical children and those with ADHD, but there was no significant effect of choice on enjoyment (Kakoulidou et al., 2021). This adds to the evidence that using self-report to measure affective responses to situational triggers in young children may be unreliable. Methods sensitive to attentional change, such as eye-tracking or event-related potential or measurement of brain activity (electroencephalogram (EEG) or MRI) may clarify the mechanisms at play.

It matters how choice is implemented. Previous findings for the effectiveness of choice have been inconsistent (e.g., Flowerday & Schraw, 2000; Patall, 2013): this study provides empirical support for the value of choice and highlights characteristics that are necessary for choice to function as a motivational tool. Findings indicate that choice needs to be meaningful (that is, offer opportunities for personal investment and autonomy) and may perform successfully with as few as two choices. Making use of situational interest in the classroom, teachers need to manage choice, by using techniques such as offering them appropriately levelled books. Programmes such as CORI offer structures for choice, other aspects of situational interest, and for motivation more widely.

In contrast to prior studies, this study provides empirical support for the effectiveness of choice as a motivational tool for young children regardless of ability or gender (e.g., deSousa & Oakhill, 1996; Schraw et al., 2001). There is a shortage of research on the impact of situational triggers in this age group (Guo & Fryer, 2024), although it is a key age for comprehension development (Hirsch, 2003) and for establishing reading habits and attachment to reading (Cremin & Scholes, 2024). Notwithstanding, it is important to recognise that these findings may be limited in wider relevance insofar as our understanding of the developmental progress of motivation and interest is rudimentary. Furthermore, although a good sample size, the participants were drawn from only four classes in two schools, making it non-viable to explore school effects.

This study is also limited by the low level of internal consistency for one of the reading comprehension measures and the correlations with NGRT raw score. It is possible that the mix of literal and inferential question type affected the homogeneity of the measure which included only 12 items. It has been suggested that alphas provide a conservative estimate of reliability. Whilst stronger consistency is desirable, it is worth noting that poor internal consistency typically undermines the likelihood of finding significant effects due to increased error (Pedhazur & Schmelkin, 1991) and this study found significant effects for the manipulation with medium effect size.

## Conclusion

As one of the few studies directly investigating the processes that mediate the links between intrinsic motivation and reading comprehension performance, this study provides empirical evidence for the effective role of choice as a trigger for situational interest and motivational tool for young children. There is an assumption that motivation impacts reading attainment but the evidence is often constrained by methodological challenges in how motivation is measured and the presence of confounding variables, such as background knowledge or a composite approach in interventions. The present study is important because it addresses these issues and demonstrates the potentially powerful effect of carefully managed choice where a small manipulation can have a strong effect. These findings indicate that sustained manipulation of situational interest may have considerable implications in practical teaching applications and that future investigations into other potential triggers, and how they can be translated into personal interest, would merit further research.

## Data Availability

Data available on request.

## Appendix A

Table showing School Characteristics for participating schools at time of testing.

**Table:**

	School I	School II
Type of School	Primary (ages 4–11)	Junior (ages 7–11)
Number of pupils on roll	425	241
Number of Pupil Premium^a^ pupils	Below national average	Below national average
Number of SEN pupils	Below national average	Above national average
Number of ESL pupils	Below national average	Above national average
Ofsted^b^ Rating	Good	Outstanding

Data provided by both schools showed that, at the time of testing, 45.5% (*N* = 50) of pupils tested spoke English as a second language (ESL) but that only 0.9% (*N* = 1) was at an early stage of learning English, 13% (*N* = 14) of pupils were classified as Pupil Premium and 13% (*N* = 14) were assessed as having special educational needs (SEN)

^a^measure of social disadvantage

^b^Office for Standards in Education

## Appendix B

Sample of statements from Enjoyment Questionnaire.

I really enjoyed this story. 1 2 3 4.

I was interested in what was going to happen. 1 2 3 4.

I found it difficult to keep going. 1 2 3 4.

The questions were easy. 1 2 3 4.

## Code availability

N/A.

## Declarations

This paper draws on the PhD thesis of the lead author, Lisa Fridkin, which is available at https://discovery.ucl.ac.uk/id/eprint/10053870/. However, statistical analysis and literature review have been significantly reviewed.
